# Abuse and dependence potential of sphingosine-1-phosphate (S1P) receptor modulators used in the treatment of multiple sclerosis: a review of literature and public data

**DOI:** 10.1007/s00213-021-06011-6

**Published:** 2021-11-13

**Authors:** Kerri A. Schoedel, Carine Kolly, Anne Gardin, Srikanth Neelakantham, Kasra Shakeri-Nejad

**Affiliations:** 1 Altreos Research Partners, Inc, Toronto, Canada; 2 Novartis Institutes for Biomedical Research, Novartis Pharma AG, Basel, Switzerland; 3 Novartis Institutes for Biomedical Research, Novartis Healthcare Pvt Ltd, Hyderabad, India

**Keywords:** Abuse, Clinical, Dependence, Fingolimod, Non-clinical, Ozanimod, S1P receptor modulator, Siponimod

## Abstract

Abuse and misuse of prescription drugs remains an ongoing concern in the USA and worldwide; thus, all centrally active new drugs must be assessed for abuse and dependence potential. Sphingosine-1-phosphate (S1P) receptor modulators are used primarily in the treatment of multiple sclerosis. Among the new S1P receptor modulators, siponimod, ozanimod, and ponesimod have recently been approved in the USA, European Union (EU), and other countries. This review of literature and other public data has been undertaken to assess the potential for abuse of S1P receptor modulators, including ozanimod, siponimod, ponesimod, and fingolimod, as well as several similar compounds in development. The S1P receptor modulators have not shown chemical or pharmacological similarity to known drugs of abuse; have not shown abuse or dependence potential in animal models for subjective effects, reinforcement, or physical dependence; and do not have adverse event profiles demonstrating effects of interest to individuals who abuse drugs (such as sedative, stimulant, mood-elevating, or hallucinogenic effects). In addition, no reports of actual abuse, misuse, or dependence were identified in the scientific literature for fingolimod, which has been on the market since 2010 (USA) and 2011 (EU). Overall, the data suggest that S1P receptor modulators are not associated with significant potential for abuse or dependence, consistent with their unscheduled status in the USA and internationally.

## Introduction

Prescription drug misuse and abuse are major public health problems in the USA and other countries. While opioids and tranquilizers (such as benzodiazepines) account for most of the prescription drug abuse, as more opioid products are being re-formulated with abuse-deterrent properties, some individuals who abuse prescription drugs may be switching to other central nervous system (CNS)-activating drugs that are not controlled, such as gabapentin (Evoy et al. 2019). The US Food and Drug Administration (FDA) recently approved several new centrally active drugs, such as ozanimod and ponesimod, for the treatment of relapsing forms of multiple sclerosis (MS), including clinically isolated syndrome, relapsing–remitting disease, and active secondary progressive disease, in adults (Zeposia Prescribing  Information, 2020 and Ponvory Prescribing Information, 2021) earlier, in March 2019, siponimod was approved by the FDA for the same indication (Mayzent Prescribing Information, 2019) and is the first treatment to demonstrate a clinically relevant effect on disability progression in the secondary progressive MS population. Sphingosine-1-phosphate (S1P) receptor modulators induce long-lasting internalization of S1P_1_ receptors expressed on the lymphocytes, inhibiting the egress of lymphocytes from lymph nodes, and preventing infiltration of autoreactive lymphocytes into the CNS, thereby reducing neuroinflammation (Gergely et al. 2012). Non-clinical evidence suggests that S1P modulators regulate neuro-inflammatory processes and may promote CNS repair mechanisms and remyelination (Gentile et al. 2016). Ozanimod, ponesimod, and siponimod share pharmacological similarities with another approved drug fingolimod as well as other S1P receptor modulators in development (e.g., amiselimod). Other members of this class have also been studied for use in other conditions, including chronic inflammatory demyelinating polyradiculoneuropathy, amyotrophic lateral sclerosis and renal transplants (fingolimod), chronic plaque psoriasis (ponesimod), ulcerative colitis (amiselimod and etrasimod), systemic lupus erythematosus (cenerimod), and other autoimmune diseases, such as Crohn’s disease, atopic dermatitis, and alopecia areata.

According to the FDA Guidance, all CNS-active new drugs must be assessed for their abuse and dependence potential (Center for Drug Evaluation and Research (CDER) 2017). However, fingolimod, siponimod, ponesimod, and ozanimod do not have any information in Sect. 9 (“Drug Abuse and Dependence”) of their US prescribing information. This is consistent with the recent FDA draft guidance indicating that Sect. 9 applies to drugs that are controlled under the Controlled Substances Act (CSA) or those that are “not controlled under the CSA for which there is important information to convey to health care providers related to abuse and dependence” (Center for Drug Evaluation and Research (CDER) 2019a). The absence of Sect. 9 in the US labels of these drugs suggests that the FDA believes that there is no information to convey regarding the abuse or dependence potential of fingolimod, siponimod, ponesimod, or ozanimod; however, the data contributing to these decisions are not readily available to researchers or health care practitioners.

Therefore, in this article, we reviewed the available data on the abuse and dependence potential of FDA-approved S1P receptor modulators (including fingolimod, siponimod, ozanimod, and ponesimod) based on published literature and other publicly accessible sources of information (including open access regulatory review documents). This includes examination of what is known about the pharmacology of the class, animal studies on abuse and dependence potential, clinical data including pharmacokinetic factors, pharmacodynamics, and adverse event (AE) profiles that may be associated with abuse as well as a review of literature on any cases of actual abuse, misuse, diversion, dependence, or withdrawal with S1P receptor modulators (primarily fingolimod, as it has been in the market for several years). We believe that this review will help to fill an information gap left by the absence of this information in the US-based prescribing information, as prescription drug abuse is an important topic that needs to be addressed for all CNS-active drugs.

## Non-clinical pharmacology and behavioral effects in animals

Different S1P receptor modulators have slightly different neuropharmacology, with varying selectivity for S1P receptor subtypes. While fingolimod is relatively non-selective and modulates S1P_1_, S1P_3_, S1P_4_, and S1P_5_ receptor subtypes, siponimod, and ozanimod are selective for S1P_1_ and S1P_5_ receptor subtypes, and ponesimod selectively modulates the S1P_1_ receptor sub-type (Chaudhry et al. 2017; Sugahara et al. 2017). Less data are available for other S1P receptor modulators that were not yet approved at the time of manuscript review, such as amiselimod, etrasimod, and cenerimod, while some members of this class (ceralifimod and GSK2018682) have been discontinued in development, and since no relevant data were identified for these drugs, they are not discussed further.

A search of Medline, Embase, and Cochrane was performed in February 2020 using the following search parameters with limits for “animals” and “2008 to present,” and excluding humans: [Dopamine, Monoamine, Nucleus accumbens, Hypothalamus, Microdialysis, Discrimination, Discriminative, Generalization, Generalize, Self-administration, Reinforce, Reinforcement, Reinforcing, Reward, Rewarding, Conditioned Place Preference, CPP, Conditioned Place Aversion, Intracranial self-stimulation, ICSS, self-stimulation, Dependence, Withdrawal, Discontinuation, Post-treatment OR Rebound] AND [Siponimod, Fingolimod, Ponesimod, Ozanimod OR Sphingosine-1-Phosphate]. No published animal abuse or dependence potential studies with S1P receptor modulators were identified in the scientific literature. However, some data are available in the FDA review material for siponimod, ozanimod, and ponesimod (Center for Drug Evaluation and Research (CDER), 2019b; Center for Drug Evaluation and Research (CDER), 2020b; Center for Drug Evaluation and Research (CDER) 2021).

Non-clinical studies performed during development of siponimod and ozanimod included in vitro and in vivo studies (Center for Drug Evaluation and Research (CDER), 2019b; Center for Drug Evaluation and Research (CDER), 2020b). For siponimod, the FDA review documents indicate that “siponimod [at 10 µM] binds to receptors (e.g., dopamine, serotonin, and opiate-mu, -delta and -kappa) that are associated with abuse-related effects. However, due to the high levels of plasma-bound siponimod (greater than 99.9%) it is not expected that significant concentrations of siponimod will reach the brain to activate these receptors…the concentrations of siponimod at the therapeutic dose of 2 mg (and 10 mg, 5 times the therapeutic dose are estimated to be present in the cerebrospinal fluid (CSF) (presumed surrogate for brain concentrations) several thousand-fold lower than the receptor binding IC_50_ values for any of the off-target sites.” This indicates that siponimod is selective for S1P receptors and is not expected to have off-target effects known to be associated with abuse at clinically relevant concentrations. Although it is quite reasonable to use the unbound drug concentration in plasma, to predict the unbound drug concentration at the active site in the brain provided that there is no active transporter involved in drug distribution (free drug hypothesis) (Lin, 2008), several additional factors may also influence brain exposure, such as protein binding in brain tissue, physicochemical properties, and brain disposition.. The FDA review documents for ozanimod indicate that “Binding assays demonstrate that ozanimod and its metabolite CC1084037 bind to several targets associated with abuse-related effects”; however, these findings were not confirmed in vivo, suggesting that off-target activities are not clinically relevant. According to the FDA review documents, ponesimod “does not bind to abuse-related targets” (Center for Drug Evaluation and Research (CDER) 2021). Although S1P receptor modulators may not directly bind to abuse-related receptors, in vitro studies published in the literature have suggested that exogenously applied S1P appears to modulate glutamatergic pathways, primarily through the regulation of glutamate secretion (Riganti et al. 2016; Sim-Selley et al. 2009), and may also affect the release of gamma-aminobutyric acid (GABA) (Kanno and Nishizaki 2011), both pathways that are associated with abuse (i.e., N-methyl-D-aspartate [NMDA] antagonists such as ketamine and GABAergic drugs such as barbiturates and benzodiazepines). This appears to be mediated primarily by the S1P_3_ receptor sub-type (Kanno and Nishizaki 2011; Riganti et al. 2016). However, glutamate levels are higher in the cerebrospinal fluid and brain tissue of MS patients, and NMDA and kainate receptors are upregulated (Newcombe et al. 2008; Srinivasan et al. 2005). Thus, modulation of the central S1P pathways could correct cortical excitability in these patient populations, as indicated by a recent clinical study with fingolimod (Landi et al. 2015), rather than affecting normal glutamatergic activity in healthy individuals.

Three animal studies with siponimod evaluated different aspects of abuse and dependence potential (Center for Drug Evaluation and Research (CDER), 2019b). A drug discrimination study in rats found no subjective similarities (“discriminative stimulus effects”) between siponimod and midazolam (a prototypical sedative) and amphetamine (a prototypical stimulant), indicating that siponimod does not have subjective effects similar to these drug classes of abuse. A self-administration study found that rats trained to self-administer cocaine did not self-administer siponimod at rates higher than the vehicle, demonstrating that siponimod does not have reinforcing effects in animals. Finally, a 28-day chronic dosing rat physical dependence study compared siponimod with vehicle and diazepam as a positive control. In contrast to diazepam, no differences were observed in withdrawal-related behavior and signs in rats who were administered siponimod compared with those administered vehicle. These data indicate that chronic exposure to siponimod does not induce physical dependence in animals. A single animal abuse study was performed with ozanimod and its major active metabolite CC112273. This self-administration study did not demonstrate any reinforcing effect of ozanimod or CC112273 in rats (Center for Drug Evaluation and Research (CDER), 2020b). No animal drug discrimination or physical dependence studies with ozanimod or CC112273 were reported. According to the FDA review material, no dedicated animal abuse potential studies were conducted with ponesimod; however, it was noted that ponesimod “does not induce acute CNS- and discontinuation/withdrawal-related symptoms or behaviors in animals” (Center for Drug Evaluation and Research (CDER) 2021).

While no relevant non-clinical data on other S1P receptor modulators were identified at the time of this review, the non-clinical data outlined in regulatory review documents indicate that there are no signals associated with abuse and dependence potential for this class.

## Clinical pharmacology data relevant to abuse

A search of Medline, Embase, and Cochrane was performed in February 2020 using the following search parameters with limits for “humans” and “2008 to present”: [addiction, psychosis, aggression, agitation, amnesia, anxiety, cognitive impairment, balance disorder, confusion, déjà vu, delirium, delusion, disorientation, dizziness, drunk, dysphoria, euphoric, euphoria, hallucination, illusion, memory, panic attack, paranoia, psychotic, sedation, somnolence, liking OR subjective] AND [siponimod, fingolimod, ponesimod, ozanimod OR Sphingosine 1-Phosphate]. The articles retrieved from these searches were also evaluated for AEs potentially associated with abuse as described in the next section.

No human abuse potential studies in recreational drug users or other clinical studies of subjective/abuse-related effects were identified for any S1P receptor modulator. The FDA review documents for siponimod, ponesimod, and ozanimod indicate that human abuse potential studies were not considered necessary based on the non-clinical and clinical AE data (Center for Drug Evaluation and Research (CDER), 2019b; Center for Drug Evaluation and Research (CDER), 2020b; Center for Drug Evaluation and Research (CDER) 2021). However, clinical data are available for pharmacokinetic factors, which may have an impact on the abuse potential of drugs of abuse, such as opioids or stimulants, for which the more rapid onset and elimination are known to be associated with a greater “high” and abuse potential. Table 1 summarizes available pharmacokinetic data for the active moieties of S1P modulators (i.e., parent [if active] and active metabolites). Note that fingolimod has no pharmacological activity and undergoes phosphorylation to produce fingolimod-phosphate (fingolimod-P), the active moiety. As shown in Table 1, most S1P receptor modulators have a moderately slow or very slow onset (time to maximum plasma concentrations of 2.5 to 4 h for siponimod and ponesimod and 6 to 21 h for fingolimod-P and ozanimod/CC112273 [the combination of which is thought to be the active moiety of ozanimod]) and moderately long or very long elimination half-lives (17 to 36 h for siponimod, ozanimod, and ponesimod and ≥ 168 h for fingolimod-P and the major active metabolite of ozanimod [CC112273]), which may partly contribute to the lack of abuse-related subjective effects for this class (as described in the next section). In addition, in available data (fingolimod, ponesimod, and siponimod), the unbound fractions of drug (free circulating drug) are fairly low (due to high plasma protein binding), which may contribute to receptor selectively for S1P and lack of off-target effects on abuse-related molecular targets as described in the previous section (Boehler et al. 2017; David et al. 2012; Gardin et al. 2017; Gergely et al. 2012; Glaenzel et al. 2018; Guerard et al. 2016; Hoch et al. 2014; Rasche and Paul 2018; Shakeri-Nejad et al. 2015; Tran et al. 2018; Center for Drug Evaluation and Research (CDER) 2010a, 2019b, 2020a, b).

**Table 1:** Summary of pharmacokinetic parameters of S1P receptor modulators (active moieties)

Drug/active metabolite(s)	Median T_max_ or T_max,ss_ (hours)	Absolute bioavailability (%)	Mean T_1/2_ (hours)	fu (%)	References
Fingolimod-P (active)^a^	6	NA	168	0.3	David et al. 2012; CDER 2010a
Siponimod	3–4	84	22–36	0.017–0.055	Gergely et al. 2012; CDER 2019b; Gardin et al. 2017
Ozanimod	6–8	-	20	1.8	CDER 2020a; Tran et al. 2020
CC112273 (major active moiety)	6–10	NA	280	0.2	
CC1084037	16	NA	280	0.7	
Ponesimod	2–4	83.8	33	0.4	CDER 2020c

^a^Fingolimod has no pharmacological activity and undergoes phosphorylation to produce fingolimod-P, the active moiety

*fu* unbound fraction; *NA* not applicable; *S1P* sphingosine-1-phosphate; *T*_max_ time to maximum plasma concentration; *T*_max,ss_ time to maximum plasma concentration at steady-state; *T*_1/2_ elimination half-life

## Clinical AE data suggestive of abuse or dependence potential

According to the FDA Guidance (Center for Drug Evaluation and Research (CDER), 2017), CNS AEs that are potentially “abuse-related” should be systematically evaluated. These AEs may signal subjective effects of interest to individuals who abuse drugs, such as mood-elevating (e.g., “euphoric mood”), hallucinogenic, sedative (e.g., “somnolence” or “sedation”), or stimulant (e.g., “psychomotor hyperactivity”) effects (Mansbach et al. 2010; Sellers and Romach 2018).

### Clinical trial data from the scientific literature

Published clinical trials were reviewed to extract psychiatric system or nervous system disorders potentially associated with abuse (according to the FDA Guidance; Center for Drug Evaluation and Research (CDER), 2017). Although there are numerous additional trials with S1P receptor modulators, particularly for fingolimod, many articles did not report the AEs of interest for this review and therefore have not been cited. Data are presented as proportion of subjects reporting the AE (percent incidence) with active drug along with placebo-corrected incidence (shown as percent incidence with active drug − percent incidence with placebo) where placebo data were available. Ranges of data (i.e., X% to X%) represent percent incidence (and placebo-corrected percent incidence) reported across individual studies; no pooling was performed. A review of published clinical trials of S1P receptor modulators demonstrated that the most common potentially CNS-related AEs associated with this class are dizziness and fatigue/tiredness, with a lower incidence of other AEs of interest, such as somnolence (indicating sedative effects), anxiety, or depression (Table2). Only 1 trial reported other events of interest (“euphoric mood” for ponesimod). Although dizziness is listed as a “euphoria-related” term of interest in the FDA Guidance (Center for Drug Evaluation and Research (CDER), 2017), in this case it may be related to the well-known transient bradyarrhythmic and heart-rate decreasing effects of this class (Urbano et al. 2013) rather than being a direct CNS effect that may indicate euphoria. As shown in Table 2, although the incidence of dizziness was higher with ponesimod compared with the other members of the class, it may simply be an artifact of the larger number of healthy subject trials published for this drug (which tend to have small sample sizes that skew incidence data and are often open-label) and the more complete AE data provided in the articles. Indeed, the incidence of dizziness is more similar for trials in relapsing–remitting MS (RRMS) patients. Some AEs that potentially indicate centrally mediated effects, such as depression and anxiety, have also been reported; however, MS patients frequently present with anxiety and depression, i.e., these have been reported in over 20% of MS patients (Marrie et al. 2015). In several studies and 1 meta-analysis, fingolimod and other disease-modifying therapies (DMTs) for MS had no significant association with the onset of anxiety, depression, or other neuropsychiatric events (Al-Hussain et al. 2017; Gasim et al. 2018; Moreau et al. 2017; Tauil et al. 2018). In a subset of 141 African-American RRMS patients from a randomized open-label study, fingolimod (0.5 mg/day) was associated with a lower AE rate (AEs per patient-year) compared with injectable DMTs for anxiety (0.055 vs. 0.161) and depression (0.092 vs. 0.134) (Cascione et al. 2018). Higher rates of dizziness (0.091 vs. 0.066) and fatigue (0.114 vs. 0.065) were observed with fingolimod vs. injectable DMTs in this study and in another open-label study of RRMS patients (reported as percent incidence: 6.4% vs. 2.9% for dizziness and 11.5% vs. 5.7% for fatigue; Fox et al. 2014), although in many trials, the incidence of fatigue is similar or lower with fingolimod compared with placebo (Table2) (Al-Salama 2019; Berry et al. 2017; Biswal et al. 2015; Boehler et al. 2017; Brossard et al. 2014; Budde et al. 2002; Calabresi et al. 2014; Cohen et al. 2016; Gardin et al. 2018; Glaenzel et al. 2018; Hoch et al. 2014; Hoch et al. 2015; Hughes et al. 2018; Juif et al. 2015; Juif et al. 2017; Kappos et al. 2006; Kappos et al. 2018a; Kappos et al. 2010; Khatri et al. 2011; Laroni et al. 2014; Lublin et al. 2016; Montalban et al. 2015; Olsson et al. 2014; Ordonez-Boschetti et al. 2015; Rojas et al. 2017; Saida et al. 2012; Scherz et al. 2015; Tran et al. 2018; Vaclavkova et al. 2014).

**Table 2:** Summary of CNS adverse events of potential interest in published clinical trials

Adverse event term	Percentage of subjects/patients [percent incidence for active – percent incidence for placebo]^a^						
	Fingolimod 0.5, 1.25, and 5 mg^b^	Ponesimod 10 to 100 mg^c^		Siponimod 2 and 10 mg^d^		Ozanimod 0.25 to 3 mg^e^	
	Patients with MS	Patients with MS	Healthy subjects	Patients with MS	Healthy subjects	Patients with MS	Healthy subjects
Dizziness	0.1 to 13%	4.5 to 9.2%	0 to 64.3%	7% [+ 2%]	5.3 to 12.5%	N/R	7.4% [+ 3.2%]
	[− 3 to + 5.2%]	[+ 3.0 to + 6.7%]	[0 to + 25%]				
Fatigue/tiredness	6.5 to 33%	5.0 to 7.9%	4.2 to 50%	N/R	6.3%	N/R	5.9% [+ 1.7%]
	[− 8 to + 0.5%]	[− 0.8 to + 2.1%]	[− 27.1 to + 3.2]				
Anxiety	3.6% [0%]	N/R	N/R	N/R	6.7%	2.3–2.4%	N/R
						[+ 2.3 to + 2.4%]	
Depression	2.9 to 10%	N/R	N/R	N/R		1.2–3.4%	N/R
	[− 2.0 to + 1.1]					[+ 0.1 to + 2.3%]	
Somnolence	0.2%	N/R	15.5% [+ 3.2%]	N/R		NR	8.8% [+ 4.6%]
Euphoric mood	N/R	N/R	8.6% [+ 8.6%]	N/R		N/R	

Includes psychiatric system or nervous system disorders. Note that not all articles included coded (MedDRA) terms, so alternative terms (such as tiredness) are also reported. Unless otherwise specified, data are for patient trials, primarily in RRMS

Note that although there are numerous additional trials with S1P receptor modulators, particularly fingolimod, many did not report adverse events of interest for this review and therefore have not been cited

^a^Data are presented as percent incidence with active drug (percent incidence for active − percent incidence for placebo) where placebo data were available. Ranges of data (i.e., X% to X%) represent incidence (placebo-corrected incidence) reported across individual studies. No pooling was performed

^b^Includes patients with MS, chronic inflammatory demyelinating polyradiculoneuropathy, amytrophic lateral sclerosis, and renal transplant (Berry et al. 2017; Budde et al. 2002; Calabresi et al. 2014; Hughes et al. 2018; Kappos et al. 2006; Kappos et al. 2010; Khatri et al. 2011; Laroni et al. 2014; Lublin et al. 2016; Montalban et al. 2015; Ordonez-Boschetti et al. 2015; Rojas et al. 2017; Saida et al. 2012)

^c^Patients with MS and chronic plaque psoriasis and healthy volunteers (Boehler et al. 2017; Hoch et al. 2014, 2015; Juif et al. 2015, 2017; Brossard et al. 2014; Olsson et al. 2014; Scherz et al. 2015; Vaclavkova et al. 2014)

^d^Patients with MS and healthy volunteers (Glaenzel et al. 2018; Al-Salama 2019; Biswal et al. 2015; Gardin et al. 2018)

^e^Patients with MS and healthy volunteers (Cohen et al. 2016; Tran et al. 2018)

*CNS* central nervous system, *MedDRA* Medical Dictionary for Regulatory Activities; *MS* multiple sclerosis; *N/R* not reported; *RRMS* relapsing remitting multiple sclerosis; *S1P* sphingosine-1-phosphate

### Clinical trial data from FDA review documents

More detailed data for CNS AEs of interest in a 12-month safety population were obtained from the fingolimod FDA review documents and are summarized in Table 3. In a pooled data set of MS patients with 12 months of exposure, the most common of these AEs with 0.5 mg fingolimod (shown as fingolimod – placebo) was depression (+ 1.2% greater than placebo); however, the incidence of this event with the 1.25 mg dose was not greater than that with placebo. With the 1.25 mg fingolimod dose, dizziness and insomnia were the most common CNS AEs that occurred with a higher incidence vs. placebo (+ 1.8% and + 1.7%, respectively). In healthy subjects, dizziness occurred more frequently with fingolimod compared with placebo (incidence of 7.0% vs. 3.1% subjects with placebo; difference of + 3.9%; CDER, 2010b). Somnolence had a slightly higher incidence in fingolimod-treated subjects compared with placebo-treated subjects (0.7% vs. 0.3%; + 0.4%), while differences between fingolimod- and placebo-treated subjects were even smaller for other AEs of interest (≤ 0.2% difference).

**Table 3:** Placebo-corrected incidence of CNS adverse events of potential interest with fingolimod in pooled MS patients with 12 months of exposure from review documents

Adverse event term	PR of fingolimod – PR of placebo	
	Fingolimod 0.5 mg	Fingolimod 1.25 mg
	*N* = 854	*N* = 849
	*Ny* = 793.2	*Ny* = 746.8
Dizziness	0.8	1.7
Emotional disorder	0	0.3
Abnormal behavior	0	0.1
Feeling drunk	0	0.1
Euphoric mood	0	0.1
Mood swings	0.5	0.1
Restlessness	0.1	0.1
ADHD	0	0.1
Depersonalization	0	0.1
Emotional distress	0	0.1
Impatience	0	0.1
Mental disorder	0	0.1
Mood altered	0.6	0.1
Aggression	0.1	0.1
Derealization	0	0.1
Agitation	0.3	0
Depression	1.2	0
Irritability	0.6	0
Confusional state	0.1	− 0.2
Speech disorder	0.1	− 0.2
Cognitive disorder	0.1	− 0.3
Insomnia	-0.3	− 0.4

Modified from FDA CDER Application Number 22–527 Other Review(s)-Table 4, 2010 (CDER, 2010b) containing pooled data in MS patients with the drug exposure of 12 months

The values presented in this table are the PR with fingolimod minus the PR with placebo. *PR* = 100 patient-year rate calculated as n/Ny*100

*ADHD* attention-deficit hyperactive disorder; *CNS* central nervous system; *FDA* Food and Drug Administration; *MS* multiple sclerosis; *n* number of abuse-related adverse events that occurred to all patients; *Ny* patient-year defined as the sum of the number of days on study drug for all patients in each treatment group divided by 365.25

The FDA review documents for ozanimod indicate that “abuse-related AEs in this population [healthy subjects] were infrequent and included AEs such as somnolence, insomnia, abnormal dreams, euphoria, fatigue, decreased appetite, anxiety and energy increased and sleep abnormalities.” In a pooled data set of MS patients treated with ozanimod in phase 2 and 3 studies, the most common of these events was somnolence, occurring in 1.6% of subjects, while the other AEs occurred in ≤ 1% of subjects. In RRMS patients, the most common CNS AEs that had a higher incidence than placebo were anxiety (+ 1.6%), depression (+ 1.4%), asthenia (+ 1.3%), and fatigue (+ 1.1%). The incidence of the remaining CNS AEs was ≤ 0.5% higher vs. placebo (Table4).

**Table 4:** Placebo-corrected incidence of CNS adverse events of potential interest with ozanimod in MS patients from pooled phase 2 and 3 studies from review documents

Adverse event term	Percent incidence with ozanimod – percent incidence with placebo
	Ozanimod all doses (0.5 and 1 mg) *N* = 1944
Anxiety	1.6
Depression	1.4
Asthenia	1.3
Fatigue	1.1
Depressed mood	0.5
Anxiety disorder	0.4
Decreased appetite	0.4
Sleep disorder	0.2
Dyssomnia	0.2
Increased appetite	0.2
Memory impairments	0.2
Panic disorder	0.1
Affective disorder	0.05
Hallucinations	0.05
Mental disorder	0.05
Suicidal ideation	0.05
Suicide attempt	0.05
Somnolence	− 0.5
Disturbance in attention	− 0.9
Irritability	− 0.9
Insomnia	− 2.1

Modified from CDER 2020b

The values presented in this table are the percentage of subjects with adverse events (percent incidence) with ozanimod minus the percent incidence of adverse events with placebo

*CNS* central nervous system; *FDA* Food and Drug Administration; *MS* multiple sclerosis

A consolidated summary table of AEs was not available in the FDA review documents for ponesimod; however, data were presented by individual study. AE data from the double-blind portion of the phase 3 study in MS patients is presented in Table 5 (Center for Drug Evaluation and Research (CDER), 2021). Depression, anxiety, and somnolence were the most commonly reported CNS AEs of potential interest; however, the incidence of these types of events was similar vs. the unscheduled immunomodulator teriflunomide, with the exception of somnolence, which had a slightly higher incidence (3.2% vs. 1.6%). One subject also reported “drug withdrawal syndrome” in the ponesimod group; however, it is unclear if this was related to ponesimod or another substance, as FDA review documents indicate that “withdrawal symptoms, which may indicate physical dependence, did not occur upon discontinuation of ponesimod.” In Phase 1 studies, review document note “the occurrence of euphoric mood in four Phase 1 studies may indicate that ponesimod has abuse potential….. The occurrence of euphoric mood at supratherapeutic doses (40 mg, 75 mg) of ponesimod in some studies is further suggestive of abuse potential.” These events led the FDA to recommend further post-market monitoring for the abuse potential of ponesimod.

**Table 5:** Incidence (%) of CNS adverse events of potential interest with ponesimod from the double-blind part of the Phase 3 study in MS patients from review documents

Adverse event term	Percent incidence with ponesimod 20 mg *N* = 565	Percent incidence with teriflunomide 14 mg N = 566
Depression	3.7	5.1
Anxiety	3.2	2.8
Somnolence	3.2	1.6
Cognitive disorder	0.7	0
Disturbance in attention	0.5	0.5
Depressed mood	0.5	0.9
Depressive disorder	0.4	0
Memory impairment	0.4	0.2
Sensory disturbance	0.4	0.7
Drug withdrawal syndrome	0.2	0
Energy increased	0.2	0
Feeling abnormal	0.2	0.4
Hallucination auditory	0.2	0
Suicidal ideation	0.2	0
Amnesia	0	0.2
Lethargy	0	0.4
Affect lability	0	0.7
Agitation	0	0.2
Confusional state	0	0.2
Derealization	0	0.2
Disorientation	0	0.2
Irritability	0	0.2
Restlessness	0	0.2

Modified from CDER 2021

The values presented in this table are the incidence (%) of adverse events with ponesimod or teriflunomide—incidence (%) of adverse events with placebo

*CNS* central nervous system; *MS* multiple sclerosis

A full table of siponimod-related CNS AEs was also not provided in the FDA review documents (CDER 2019)^.^ However, some information is provided for 8 of 1333 MS patients (0.6%) who reported potentially abuse-related AEs, including feeling abnormal, derealization, hallucinations, and euphoric mood in 2 patients (0.15%) each and feeling drunk and feeling abnormal in 1 patient (0.8%) each. Most of these events were not considered by the investigators to be related to the study drug, although the assessment was not provided for a few events (feeling abnormal, derealization, and feeling drunk). In addition, the review document states that because these events were not seen in healthy subjects, it seems unlikely that siponimod caused these AEs in the MS patients.

Overall, clinical trial AE data from scientific literature and FDA review documents indicate that S1P receptor modulators do not appear, as a whole, to be associated with subjective or neuropsychiatric effects that may be of interest to individuals who abuse drugs.

## Post-market reports of actual abuse, misuse, dependence, or withdrawal

A review of literature was performed to identify any potential reports of fingolimod abuse, misuse, diversion, dependence, or withdrawal. PubMed searches were performed in February 2020 with the search term [fingolimod] AND [abuse, misuse, dependence, addict, addiction, diversion, withdrawal, overdose, intoxication, OR poisoning]. The searches were also performed for siponimod; however, due to a relatively short duration since market availability (< 1 year), cases may not yet be available in the literature.

Overall, no cases of abuse, misuse, dependence, addiction, diversion, or withdrawal syndrome related to fingolimod were identified. A single case of deliberate overdose was identified. The subject had a history of depression and had ingested a non-CNS medication in addition to fingolimod (28 fingolimod 5 mg tablets in addition to 4 phenoxymethylpenicillin 500 mg tablets). Although there is no indication that the case was related to abuse of fingolimod, it has been included for completeness, as it was not clearly stated as an attempted suicide or other self-harm attempt (Stephenson et al. 2014).

In addition, the FDA review documents for siponimod (Center for Drug Evaluation and Research (CDER), 2019b) indicate that “The Sponsor conducted searches for reports of abuse-related signals with fingolimod (Gilenya®) using publicly available post-marketing sources, including World Health Organization (WHO) VigiBase, FDA Adverse Event Reporting System (FAERS), National Poison Data System (NPDS), Drug Abuse Warning Network (DAWN, for the year 2011), scientific literature (Pubmed, up to January 2018), and Internet forums (Erowid Experience Vaults, Bluelight, and Drug Form; up to January 2018). Most searches covered a period of up to February 2017, no signals of abuse, misuse, diversion, dependence or withdrawal with fingolimod were identified.” Several cases of potential rebound or relapse of MS symptoms following discontinuation of fingolimod (Alroughani et al. 2014; Alvarez-Gonzalez et al. 2017; Beran et al. 2013; Berger et al. 2015; Czlonkowska et al. 2017; Davion et al. 2016; Faissner et al. 2015; Forci et al. 2017; Fragoso et al. 2019; Ghezzi et al. 2013; Giordana et al. 2018; Gunduz et al. 2017; Hakiki et al. 2012; Havla et al. 2012; La Mantia et al. 2014; Lapierre et al. 2016; Lapucci et al. 2019; Novi et al. 2017; Sacco et al. 2020; Sanchez et al. 2018; Sempere et al. 2013; Ward et al. 2016) and one potential case from a clinical trial involving siponimod (Litwin et al. 2018) were reported. In the latter case, disease exacerbation was reported 12 weeks after siponimod discontinuation. Recurrence of disease activity within 10 to 12 weeks of discontinuing effective MS DMT therapy is not unexpected in MS. A few non-clinical studies have identified a potential mechanism of relapse/rebound with fingolimod as being related to lymphocyte re-infiltration (Reddy et al. 2014; Yoshida et al. 2011), an effect that is observed with other immunosuppressive agents such as natalizumab. Indeed, none of the case reports referenced above contained any mention of withdrawal symptoms, other than the recurrence of the disease. In addition, the presence of a rebound effect vs. simply a relapse of the disease following fingolimod discontinuation has not been confirmed beyond individual case reports and several small retrospective chart reviews, in which rebound was suspected in 5% to 10% of the patients who discontinued fingolimod but causality cannot be confirmed (Evangelopoulos et al. 2018; Frau et al. 2018). Large retrospective analyses investigating more than 2000 patients from clinical trials of fingolimod failed to find a difference with regard to exceptionally high disease activity between previously placebo- and fingolimod-treated patients after study drug discontinuation (De-Vera et al. 2010; Vermersch et al. 2017; Vollmer et al. 2013), although the US prescribing information for fingolimod now contains a warning regarding the potential for severe increase in disability after stopping (Gilenya Prescribing Information﻿, 2018).

Although this immunologic phenomenon has no relevance to the dependence potential of fingolimod (i.e., emergence of withdrawal symptoms, which may lead to the continued use of a drug beyond the time that it is medically indicated), it is nonetheless important from a clinical management perspective that any discontinuation of DMTs should be accompanied by careful monitoring for relapse or potential rebound effects.

Overall, no cases of fingolimod abuse, misuse, addiction, dependence, diversion, or withdrawal syndrome were identified in the scientific literature. Furthermore, there is no evidence that fingolimod has any other abuse-related subjective effects or potential for psychological dependence, which would be necessary components of any potential dependence syndrome.

## Discussion and conclusions

Prescription drug abuse is a major public health problem in the USA and other countries. A few studies have found a higher incidence of substance use disorders in MS patients compared with the general population, which may be associated with risk-taking behavior as a predisposing factor to the development of MS rather than substance use after the onset of MS (e.g., higher risk of pre-symptomatic use of alcohol and recreational drugs) (Bombardier et al. 2004; Hawkes and Boniface 2014). Given the possibility of abuse of CNS-active drugs, even those that are not controlled, it is important to review the information for the new drugs, even those for relatively small and objectively diagnosed populations, such as MS patients, who may present unique risk factors. The available literature and public documents indicate that S1P receptor modulators, such as ponesimod, ozanimod, siponimod, fingolimod, and those in development, are not likely to be abused. Although it is possible that the lack of reports of actual abuse or misuse of fingolimod, which has been marketed for more than 10 years, are related to under-reporting due to a lack of awareness of the issue, the limited non-clinical data and extensive AE reports suggest that this class does not have properties that would make them attractive for abuse, such as mood-elevating, sedative, stimulant, or hallucinogenic effects. One limitation of the AE data obtained from the literature is that many articles only presented more common AEs, typically those occurring in > 5% or 10% of the patients; therefore, less frequently observed CNS AEs may not have been reported. Although it is suggested that some AEs of potential interest for abuse may not have been reported, the fact that these AEs are not common enough to be reported at the 5% or 10% threshold lends credence to the idea that these drugs do not produce CNS effects frequently enough to be of interest to abusers. This was confirmed in the FDA review documents for ponesimod, fingolimod, siponimod, and ozanimod, in which CNS AEs were reported relatively infrequently, the most common of which were dizziness and possibly insomnia, along with other neuropsychiatric AEs, such as anxiety, depression, or fatigue/asthenia, which are not directly related to abuse potential. While ponesimod was associated with cases of “euphoric mood” in phase 1 studies in healthy subjects that were apparently not seen with other members of this class, it is unclear whether this will be associated with abuse in the post-market setting. Notably, some of the phase 1 studies in question were open-label, and therefore the data are difficult to interpret in the absence of a placebo control. In addition, this class of drugs is associated with events that may be aversive and further deter any potential for experimentation, such as headache, diarrhea, liver transaminase elevation, cough, influenza, sinusitis, and pain (Vollmer et al. 2013).

Finally, this review was based on public literature and data sources and does not comprehensively review all potential data sources for the identification of potential abuse signals, such as FAERS and EudraVigilance or other post-marketing surveillance systems. Such evaluations would be beyond the scope of this review. In addition, to our knowledge, no human abuse potential study considered as the gold standard for evaluating the potential for recreational abuse of a drug has been conducted with a member of this class and no systematic evaluation of physical dependence in humans is available. Although no data suggest that S1P receptor modulators are associated with a withdrawal syndrome similar to other DMTs, a potential for recurrence of the disease exists, and although unproven, rebound activity should be considered.

Despite the limitations of the available data, S1P receptor modulators, such as fingolimod, siponimod, ponesimod, and ozanimod, as well as similar drugs in development, assuming that they show selectivity for S1P receptors, do not appear to be associated with abuse or dependence potential, and no signals of abuse or dependence were identified in the literature or other public documents.
